# Cathodoluminescence differentiates sedimentary organic matter types

**DOI:** 10.1038/s41598-024-53168-z

**Published:** 2024-03-12

**Authors:** Paul C. Hackley, Ryan J. McAleer, Aaron M. Jubb, Brett J. Valentine, Justin E. Birdwell

**Affiliations:** 1 U.S. Geological Survey, Geology, Energy & Minerals Science Center, Reston, VA 20192 USA; 2 U.S. Geological Survey, Florence Bascom Geoscience Center, Reston, VA 20192 USA; 3 U.S. Geological Survey, Central Energy Resources Science Center, Denver, CO 80225 USA

**Keywords:** Precambrian geology, Stratigraphy, Petrology, Sedimentology, Environmental impact, Palaeoclimate

## Abstract

High-resolution scanning electron microscopy (SEM) visualization of sedimentary organic matter is widely utilized in the geosciences for evaluating microscale rock properties relevant to depositional environment, diagenesis, and the processes of fluid generation, transport, and storage. However, despite thousands of studies which have incorporated SEM methods, the inability of SEM to differentiate sedimentary organic matter types has hampered the pace of scientific advancement. In this study, we show that SEM-cathodoluminescence (CL) properties can be used to identify and characterize sedimentary organic matter at low thermal maturity conditions. Eleven varied mudstone samples with a broad array of sedimentary organic matter types, ranging from the Paleoproterozoic to Eocene in age, were investigated. Sedimentary organic matter fluorescence intensity and CL intensity showed an almost one-to-one correspondence, with certain exceptions in three samples potentially related to radiolytic alteration. Therefore, because CL emission can be used as a proxy for fluorescence emission from sedimentary organic matter, CL emission during SEM visualization can be used to differentiate fluorescent from non-fluorescent sedimentary organic matter. This result will allow CL to be used as a visual means to quickly differentiate sedimentary organic matter types without employing correlative optical microscopy and could be widely and rapidly adapted for SEM-based studies in the geosciences.

## Introduction

Scanning electron microscopy (SEM)-based characterization of sedimentary organic matter is widely utilized in climate and paleoclimate research^1,2^, soil^3,4^ and atmospheric science^5^, sediment contaminant studies^6^, and carbon mineralization work^7^. In energy science, the landmark description of porosity in sedimentary organic matter observed by SEM visualization of ion-milled samples of Barnett Shale^8^ resulted in a paradigm-shifting focus on the fluid transport and storage properties of petroliferous mudstones, where SEM visualization is now broadly utilized for understanding fluid generation^9–11^, fluid migration^12^, and reservoir storage properties^13,14^. Such studies are relevant for carbon sequestration^15^ and seal integrity^16^ for natural petroleum reservoirs and energy gas storage (e.g., H_2_, CH_4_). However, because of low atomic density, all sedimentary organic matter types appear similar (black) in electron microscopy^17–19^. Therefore, identification of sedimentary organic matter types via SEM is based solely, and sometimes incorrectly, on textural appearance, which has impeded the rates of learning and innovation for petrographic research on fine-grained sedimentary rocks. The inability of SEM to distinguish organic matter types may explain, in part, why thousands of SEM-based studies of organic porosity have so far not delivered definitive answers on the type of organic matter in which organic porosity forms or the precise thermal regime of its development. Loucks and Reed^20^ provided SEM-based textural criteria which can be used to distinguish secondary migrated organic matter from primary organic matter, such as the presence of migrated organic matter occurring in mineral pores subsequent to cementation. However, others have asserted that even with well-documented SEM textural criteria for sedimentary organic matter differentiation, misidentification and misinterpretation can occur. For example, the embayment of sedimentary organic matter by euhedral terminations of mineral cement is a commonly cited textural criterion for migrated organic matter, but this texture is also present in primary organic matter types deposited contemporaneously with sediments^21^. Therefore, utilization of additional tools and detection systems such as CL during SEM-based visualization is necessary for identification of sedimentary organic matter.

Organic matter fluorescence requires delocalized electrons in aromatic or conjugated moieties (fluorophores) which present with highest emission intensity when diluted in an otherwise hydrogen-rich aliphatic matrix^22^, as occurs in the liptinite group of sedimentary organic matter^23^. At higher thermal maturities, increased cross-linking polymerization and condensation effectively increases aromatization in sedimentary organic matter^24^. However, a decrease in fluorescence emission intensity arises due to a concentration quenching effect from increased molecular aromatization, which increases fluorophore density^22,25,26^. Optically observed fluorescence of sedimentary organic matter is driven by the promotion of electrons to excited energy states through photon absorption followed by photon emission during relaxation of the excited electrons, whereas CL is a similar process driven by electron absorption, i.e., electron-induced light emission. Organic compound CL is employed in pharmaceutical research^27^ and CL is a mature area of investigation for inorganic diagenesis of sedimentary rocks including organic-rich mudstone^28–30^, where CL is primarily used to investigate mineral reactions which occur during burial diagenesis. Cathodoluminescence detectors are a commonly installed component of SEM systems, yet, to our knowledge, the CL emission of sedimentary organic matter has been explored in only a few studies^31,32^. The optimized operating conditions to limit beam damage to sensitive organic materials are unknown, and we are unaware of any work that has utilized CL emission to identify individual sedimentary organic matter types.

Herein, we used a CL detector installed in a SEM to visually document the CL properties of different sedimentary organic matter types. Using correlative reflected light, fluorescence, secondary electron, and CL images, and Raman spectroscopy, we describe CL emission of different sedimentary organic matter types in mudstone from low thermal maturity conditions. The results show that CL emission is similar to fluorescence from traditional optical microscopy (with certain exceptions), and is inversely related to reflectance and Raman spectroscopy properties, e.g., first-order Raman carbon D1- and G-band peak intensities. This result will potentially allow CL emission to be utilized as a visual aid for sedimentary organic matter identification during routine SEM imaging of similar thermal maturity sedimentary rocks in geosciences research.

## Results

### CL and fluorescence emission intensity comparison

**Figure 1:**
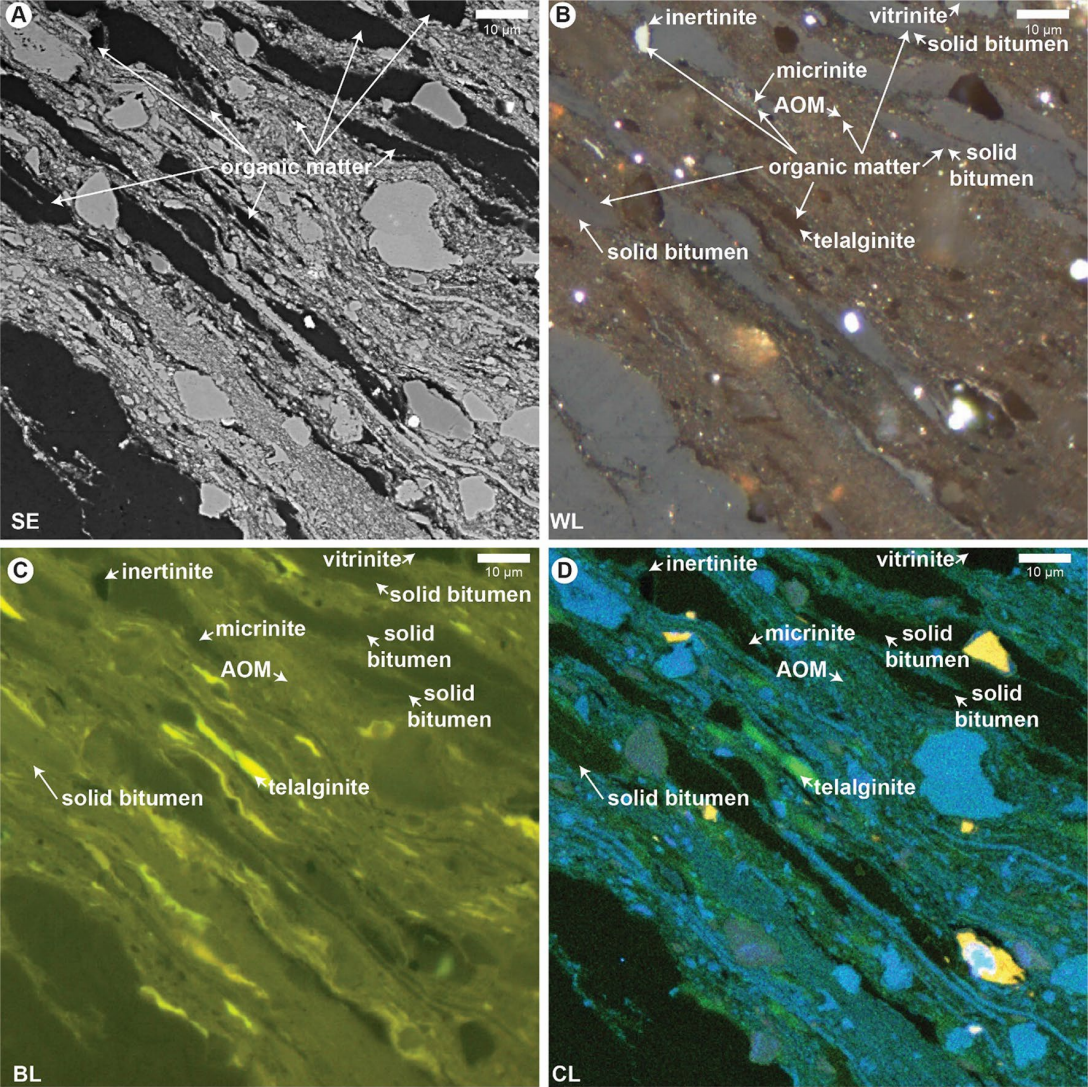
Sedimentary organic matter types in the Lower Cretaceous Sunniland Limestone. (**A**) Secondary electron (SE) image. (**B**) Same field as A under white light (WL). (**C**) Same field as (**A**–**B**) under blue light (BL) fluorescence. (**D**) Same field as (**A**–**C**) under cathodoluminescence (CL). AOM, amorphous organic matter.

**Figure 2:**
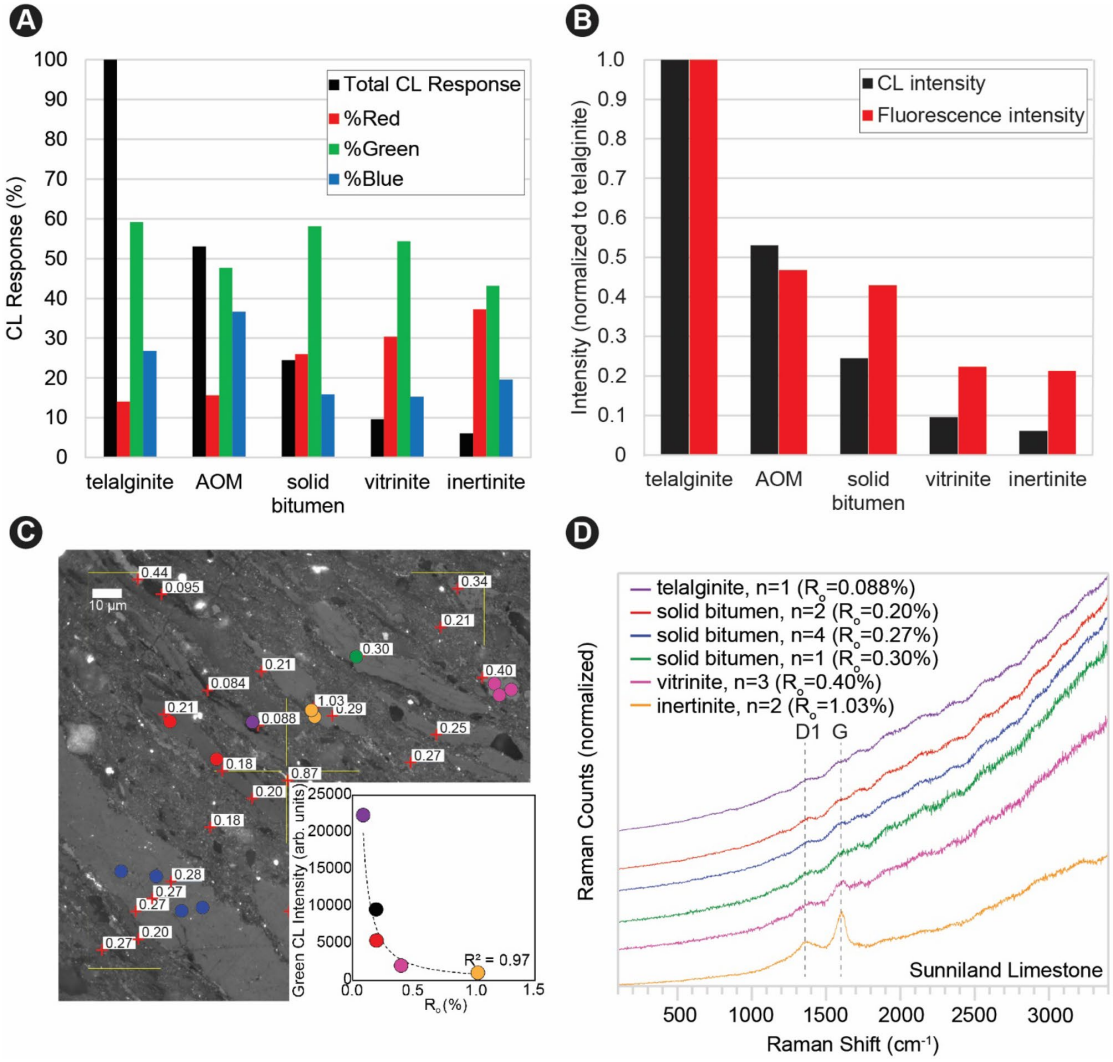
Color analysis, reflectance, and Raman spectroscopy of sedimentary organic matter types in the Lower Cretaceous Sunniland Limestone. (**A**) CL emission (normalized to telalginite) and RGB percentages of emission. (**B**) CL intensity and fluorescence intensity (both normalized to telalginite emission). (**C**) Reflectance values (in monochrome image) and co-located Raman measurements. Locations of Raman measurement indicated by colored circles, with color corresponding to spectral traces in panel (**D**). Lower right inset shows CL emission intensity vs. reflectance with colored circles corresponding to different organic matter types (black is amorphous organic matter). (**D**) Raman spectra with peak centers corresponding to the aromatic carbon D1 and G peaks indicated by dashed vertical lines.

We found fluorescence emission intensity and CL emission intensity (primarily in the green channel at 505–575 nm) had an almost one-to-one visually estimated correspondence for individual sedimentary organic matter types. Figure 1 illustrates sedimentary organic matter types in the Lower Cretaceous Sunniland Limestone, where panel 1A shows a secondary electron image of undifferentiated sedimentary organic matter which cannot be identified by SEM alone. Organic matter types are identified by reflectance, color, and morphology using optical microscopy in Fig. 1B, and include telalginite, amorphous organic matter (AOM), solid bitumen, vitrinite, micrinite, and inertinite. Fluorescence (Fig. 1C) confirms the sedimentary organic matter type identities and shows a qualitative decrease in fluorescence emission intensity from telalginite > AOM > solid bitumen > vitrinite > inertinite occurring in the same microscope field. Viewed using CL (Fig. 1D), organic CL emission intensity corresponds qualitatively to fluorescence intensity (cf. Fig. 1C and D). Image analysis confirms quantitatively that total CL emission intensity decreases for sedimentary organic matter types in the same order as fluorescence intensity decreases (Fig. 2A). Figure 2A also shows that the greatest proportion of CL emission is from the green channel, and that the total intensity decrease is accompanied by increasing red shift^26^ [increased proportion of emission in the red (> 605 nm in this study)]. Because the intensity of SEM-CL emission correlates with fluorescence intensity (Fig. 2B), primary sedimentary organic matter types in the liptinite group (fluorescent) can be differentiated from vitrinite and inertinite group macerals (generally non-fluorescent). As some low maturity solid bitumen can display fluorescence emission^33^, its identification is more nuanced and requires some knowledge of sedimentary organic matter textural properties for an individual sample, and the comparison of relative CL intensities.

### CL emission inversely related to reflectance

**Figure 3:**
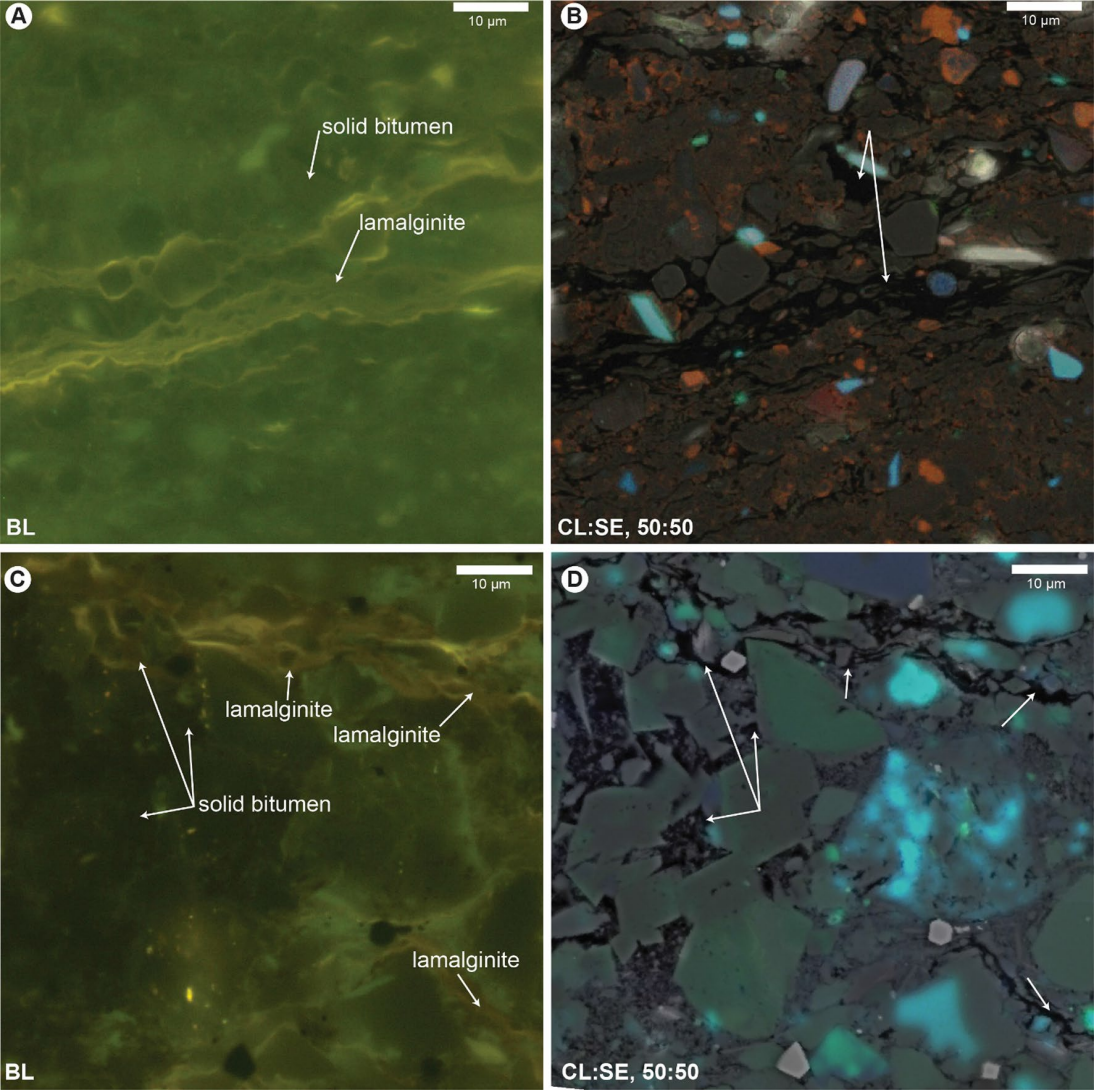
Fluorescent sedimentary organic matter with no CL emission. (**A**) Mesoproterozoic Xiamaling Formation under blue light (BL) fluorescence. (**B**) Same field as A imaged with 50:50 secondary electron (SE) and CL overlay. (**C**) Paleoproterozoic Barney Creek Formation under BL fluorescence. (**D**) Same field as (**C**) imaged with 50:50 CL:SE overlay [red channel not shown in panel (**D**) due to streaking from long lifetime luminescence from carbonates and/or apatite]. Unlabeled sedimentary organic matter in CL panels (**B**) and (**D**) is identified by fluorescence in panels (**A**) and (**C**).

Organic fluorescence and CL intensity display an inverse correlation to reflectance, i.e., as fluorescence and CL intensity of sedimentary organic matter types decrease in the Sunniland Limestone example, the reflectance of those sedimentary organic matter types systematically increases. This phenomenon is shown in Fig. 2C, where a monochrome image of sedimentary organic matter in the Sunniland shows the location and values of reflectance measurements. Organic reflectance values systematically increase in an inverse relation with CL intensity, i.e., reflectance increases from telalginite (0.084–0.095% R_o_) < AOM (0.18–0.27% R_o_) < solid bitumen (0.18–0.34% R_o_) < vitrinite (0.40–0.44% R_o_) < inertinite (1.03% R_o_). The relationship between reflectance and CL emission intensity is shown graphically in the inset to Fig. 2C, where reflectance and CL properties are inversely related in a power law expression with R^2^ = 0.97. The same inverse relation is true for Raman spectra which directly reflect organic carbon aromaticity^34^. Raman D1 and G bands for carbon could only be distinguished above fluorescence background in vitrinite and inertinite (Fig. 3D) which possess higher reflectance, although poor Raman response could be in part a function of the laser wavelength employed (see “Methods”).

## Discussion

### CL as a proxy for fluorescence

The correspondence between fluorescence emission intensity and SEM-CL emission allows CL to be used as a proxy to predict the fluorescence of sedimentary organic matter, thereby assisting with identification of individual organic matter types. Prior CL investigations of coal and industrial carbon materials did not use an imaging-based approach but attempted to assign CL emission to specific organic functional groups^31^. Organic CL emission was characterized as “*intense broadband luminescence from aromatic organic compounds*,” “*band-tail states caused by variations in the energy gap of individual sp*^*2*^* carbon clusters*,” and “*a 710 nm CL emission commonly detected also in wood and ivory, which has been correlated with hydrocarbon groups of chlorophyll or lignine*” by Kostova et al.^31^ Chen et al.^32^ presented an image of solid bitumen from Cambrian dolomite obtained by SEM-CL which unfortunately did not illustrate any CL properties of the sedimentary organic matter, i.e., the solid bitumen showed little or no CL emission. To our knowledge, CL emission has not been used to identify individual sedimentary organic matter types through an image-based approach, apart from the work presented herein.

### Causes of CL emission loss

Despite the relationship between fluorescence and CL emission noted above, we found certain sedimentary organic matter examples with CL emission loss, even in the presence of strong co-located fluorescence emission. For example, sedimentary organic matter possessing high fluorescence intensity in the Mesoproterozoic Xiamaling Formation (Fig. 3A) and in the Paleoproterozoic Barney Creek Formation (Fig. 3C) exhibited little or no CL emission (Fig. 3B and D, respectively). Highly fluorescent sedimentary organic matter in the Cambrian-Ordovician Alum Shale also displayed no CL emission. The loss of CL emission in samples which still exhibit strong organic fluorescence is here speculatively attributed to radiolytic alteration. Radiolytic alteration of sedimentary organic matter is a function of radiation intensity, duration, degree of dissemination of radioactive sources, organic matter density, and the abundance of water^35,36^. While there is no clear relationship between radiation dose (α-particle fluence calculated from bulk rock U and Th content, Table 1) and CL emission loss in our samples, we suggest that incorporation of U into the sedimentary organic matter of certain samples may have caused increased polymerization and therefore decreased CL emission from quenching effects. Alternatively, proximity to radioactive minerals in the microscope fields selected for this study may be responsible for CL emission loss in certain samples. The quenching effect to fluorescence emission from organic polymerization has been long noted in spherical ‘thucholite’ halos surrounding radioactive minerals, including in the Alum^37^, Salt Range^38^, Xiamaling^39^, and Barney Creek^40^ rocks studied herein. Nevertheless, Neoproterozoic-Lower Cambrian Salt Range samples displayed prominent CL emission from fluorescent algal and amorphous organic matter, and CL emission was present in the fluorescent margins of Salt Range thucholites. In short, the cause of CL emission loss in certain samples with strong co-located fluorescence emission is undetermined at present, and could also be attributed to a priori differences in the populations of luminescent molecular structures in different organic matter types and their subsequent diagenetic alteration.

**Table 1:** Samples used for SEM-CL investigation of sedimentary organic matter and supporting data.

Sample ID	Formation	U	Th	α dose	TOC	S1	S2	S3	T_max_	HI	OI	PI	BR_o_	s.d	No
		ppm	ppm	no./mg	wt.%	mg/g	mg/g	mg/g	°C				%		
K-2	Salt Range	34.5	3.7	6.43*10^16^	36.72	10.15	220.67	2.78	430	601	8	0.04	0.34	0.07	39
Calumet Collier 34–5 13,574'	Sunniland	nd	nd	nd	25.07	8.79	113.64	2.17	423	453	9	0.07	0.36	0.05	52
APM	Green River	5.8	4.9	9.28*10^14^	22.37	2.50	195.00	3.90	439	871	17	0.01	0.33	0.03	100
KC-1	Kimmeridge	10.5	5.1	5.36*10^15^	44.10	7.63	325.06	8.04	409	737	18	0.02	0.29	0.03	13
Alum 1	Alum	14.9	8.5	2.43*10^16^	9.06	3.46	37.90	1.18	419	419	13	0.08	0.32	0.03	nd
POI-012b	Huron	15.4	11	1.89*10^16^	7.10	0.99	20.54	0.60	435	289	8	0.05	0.87*	0.08	51
EF50	Eagle Ford	3.9	1.7	1.20*10^15^	2.20	1.70	5.60	0.20	448	242	10	0.23	0.73	0.08	25
SN-5(b)	Salt Range	39.7	1.8	7.40*10^16^	27.58	8.29	133.39	2.79	431	484	10	0.06	0.49	0.04	49
XML001	Xiamaling	9.8	8	5.12*10^16^	5.75	0.53	28.99	0.43	438	457	7	0.02	0.34	0.03	25
GRNT 79–5 237.70–237.85 m	Barney Creek	4.4	11.3	2.80*10^16^	3.00	0.77	12.42	0.29	441	415	10	0.06	0.35	0.08	26
20,844 10,260.6'	Bakken	96.5	4.8	1.18*10^17^	13.69	3.55	52.18	0.31	441	381	2	0.06	0.78	0.11	100

U, uranium; Th, thorium; ppm, parts-per-million; α dose in number (no.) decay events per mg^42^; TOC, total organic carbon in wt.%; S1 (thermal distallate), S2 (pyrolyzate) in mg hydocarbons (HC)/g rock; S3 in mg CO_2_/g rock; T_max_ in °C; HI, hydrogen index in mg HC/g TOC; OI, oxygen index in mg CO_2_/g TOC; PI, production index [S1/(S1 + S2)]; BR_o_, solid bitumen mean random reflectance in %; s.d., standard deviation of BR_o_; no., number of BR_o_ measurements; nd, no data. *Previously reported as 0.80% group mean in Hackley and Ryder^50^, this is a newly reported individual measurement.

### CL relation to thermal maturity

**Figure 4:**
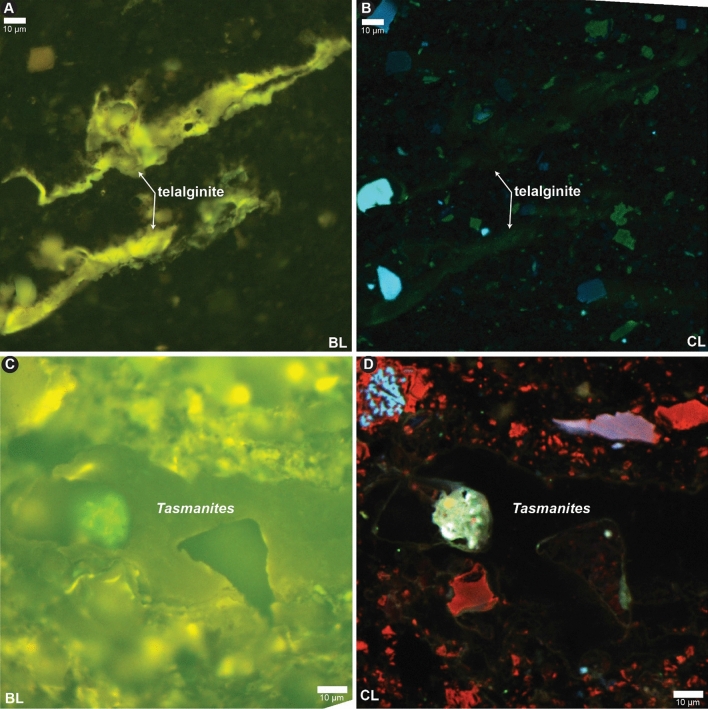
CL emission in the Bakken and Ohio Shale. (**A**) Devonian–Mississippian Bakken Formation under blue light (BL) fluorescence. (**B**) Same field as A under cathodoluminescence (CL, note red channel is not shown). (**C**) Upper Devonian Ohio Shale lower Huron Member under blue light (BL) fluorescence. (**D**) Same field as (**A**–**C**) under cathodoluminescence (CL).

CL emission is expected to diminish proportionally to loss of fluorescence emission with increasing thermal maturity. Fluorescence emission diminishes with increasing thermal maturity in sedimentary organic matter, typically below the limits of optical observation by the peak oil window^26^. We tested CL response in higher maturity samples, finding CL emission was still present in fluorescent sedimentary organic matter occurring in the peak oil window Devonian–Mississippian Bakken Formation (Fig. 4A–B). This sample has solid bitumen reflectance (BR_o_) of 0.78 ± 0.11% (Table 1), indicative of thermal conditions approaching peak oil. In addition, the moderately high bulk U content of 96.5 ppm has not apparently altered CL emission intensity. However, a similar fluorescent sedimentary organic matter type (*Tasmanites* marine alga) in the Upper Devonian Ohio Shale at slightly higher thermal conditions of 0.87 ± 0.08% BR_o_ displays no CL emission (Fig. 4C–D). This result suggests that SEM-CL differentiation of fluorescent sedimentary organic matter may be limited to peak oil conditions and below, with the upper limit for differentiation somewhere between approximately 0.8 to 0.9% BR_o_.

As documented herein, SEM-CL emission will distinguish fluorescing from non-fluorescing sedimentary organic matter. As fluorescence commonly is present in multiple types of primary organic matter within the liptinite group of organic matter^23^, and also occurs rarely in some secondary organic matter types of solid bitumen^41^, this characteristic will allow CL-based assignment of the sedimentary organic matter type broadly to fluorescent (liptinite, some solid bitumen, and minor low maturity vitrinite) or non-fluorescent categories (most vitrinite, inertinite). Therefore, CL can be used as a discriminator to be employed concurrently during SEM petrography to aid sedimentary organic matter identification during geosciences research. As we have shown herein, vitrinite and inertinite may also possess some limited CL emission, suggesting that caution and further study is required before CL can be fully established as a stand-alone tool for the SEM-based identification of sedimentary organic matter. Nevertheless, our work provides a new approach for SEM-based petrographic studies to quickly differentiate fluorescent versus non-fluorescent sedimentary organic matter types using a CL detector and without employing correlative optical microscopy. This approach is available to any SEM user with a suitable CL detector and opens a new capability for SEM-based studies of organic-rich sedimentary rocks in the geosciences.

## Methods

### Samples

Eleven organic-rich sedimentary rock samples were selected to represent a broad array of sedimentological character (clastic versus carbonate), depositional environment (lacustrine versus marine), geologic age (Paleoproterozoic to Eocene), and petrographic organic matter assemblage (vitrinite, inertinite, liptinite, and solid bitumen). Samples were from prior U.S. Geological Survey (USGS) investigations and selected specifically for the presence of fluorescent organic matter at immature to peak oil window conditions. Sample details including formation name, uranium and thorium content, programmed temperature pyrolysis and reflectance data are provided in Table 1.

### Geochemical analysis

Samples for geochemical screening analysis were crushed and ground to powder by hand using a mortar and pestle. Uranium and thorium concentrations were determined by inductively coupled plasma-optical emission spectroscopy-mass spectrometry sodium peroxide fusion at SGS or Activation laboratories with a stated detection limit of 0.1 ppm. Radiation dose (α-fluence) calculations were according to Nasdala et al.^42^. Total organic carbon (TOC) content was determined on rock powder at the USGS Petroleum Geochemistry Research Laboratory in Lakewood, Colorado, by LECO C744 carbon analyzer following carbonate removal at room temperature with 6 M HCl^43^. Programmed temperature pyrolysis of rock powder used a Hydrocarbon Analysis with Kinetics (HAWK) instrument following typical procedures^44–46^. The geochemical screening analyses were determined according to the instrument manuals and included internal laboratory standards for all runs.

### Sample preparation and optical microscopy

Crushed, homogenized sample chips of ~ 1 mm size were prepared into pelletized thermoplastic mounts via ASTM D2797^47^ using a final mechanical polish of 0.05 μm with colloidal silica. Using a Leica DM 4000 microscope, the mosaic function of the DISKUS-FOSSIL software was used to create an image map of the entire sample pellet surface with a dry 10 × (0.25 NA) objective^18^. The sample map was created as a navigation aid to return to selected regions of interest (ROI) when transferring samples between the optical, Raman, and electron microscopes. A utility knife was used to mark the pellet edge to aid with orientation. Once a ROI was identified, mean random reflectance measurements (R_o_) were collected on all organic matter types in the ROI according to a modification of ASTM D7708^48^, wherein approximately 5–20 measurements of R_o_ were collected using a 50 × (0.85 NA) oil immersion objective. R_o_ measurements were conducted on a Leica DM 4000 microscope equipped with incident white and blue LED illumination and monochrome camera light detection using the computer program DISKUS-FOSSIL from Hilgers Technisches Buero. Calibration of the microscope used a yttrium–aluminum-garnet reflectance standard (0.908% R_o_) from Klein & Becker. Following R_o_ measurement with the monochrome camera, the same microscope field was imaged in color using the white LED and in fluorescence using a blue emission LED (445 nm), a 482 nm beam splitter, and a 510 nm longpass filter. R_o_ measurements were not collected on correlative fields for all samples used in the study.

### Micro-Raman spectroscopy

All Raman spectra were collected using a Horiba Xplora Plus confocal microscope equipped with a 473 nm laser, a 50 μm pinhole, a 100 μm spectrometer slit, and a 1200 groove/mm dispersive grating. Spectra were collected using a 100 × (0.9 NA) objective, 25 or 250 μW laser power at the sample surface, 10 s acquisitions, and 3 co-adds, with assessment to assure no sample damage from the laser^49^. No thermal alteration was observed for any sample using the stated conditions. Spectra were calibrated against the 520.7 cm^−1^ response of crystalline Si and have a stated Raman shift precision of ± 1 cm^−1^.

Spectra collection workflow involved navigation to the relevant field of view using optical images and then collection of spectra from macerals where reflectance measurements had been previously collected. In some instances, multiple spectra were collected from a single organic grain and used to create a mean spectrum. Raman measurements were not collected on correlative fields for all samples used in the study.

### SEM microscopy and cathodoluminescence

Sample mounts were coated simultaneously with ~ 10 nm (estimated by quartz crystal piezometry) of carbon applied using a Leica ACE600 coater. Scanning electron microscopy was performed using a Hitachi SU-5000 field emission scanning electron microscope (FE-SEM) equipped with a Delmic SPARC modular CL detector with fast intensity mapping, operated via Odemis software. Color CL images were obtained using a filter wheel and serial collection of blue (< 505 nm), green (505–575 nm), and red (> 605 nm) channels on a photomultiplier tube (PMT) detector.

**Figure 5:**
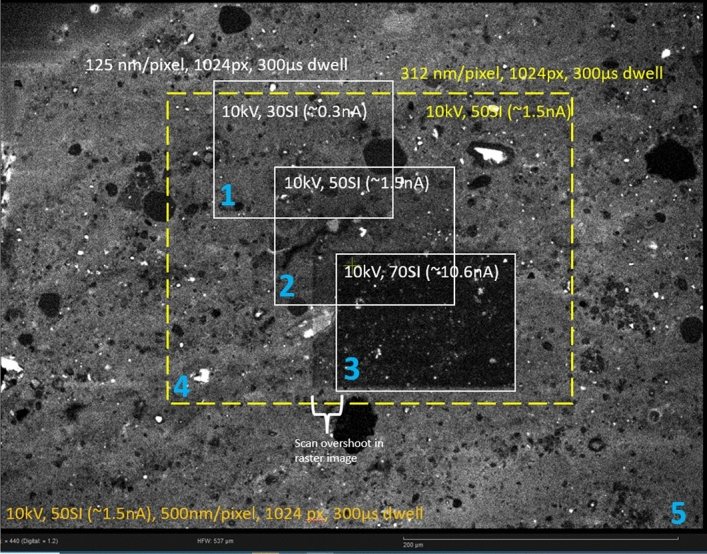
CL imaging conditions. Blue number is the order images were taken. The dashed yellow lines outline conditions for most CL acquisitions. Moving from 1 to 10 nA at 125 nm/pixel and 300 µs dwell resulted in major loss of CL signal. This example is from the Jurassic Kimmeridge Clay Formation (sample KC-1) and the illustrated field is dominated by amorphous organic matter. SI, spot intensity; px, pixel.

The CL images were collected at 10 keV and < 1nA of beam current and short dwell time to minimize damage/loss of CL signal. In RGB filtered images, the majority of the CL emission from organic matter is in the green channel (505-575 nm), but the intensity is relatively low as compared to coexisting luminescent minerals (e.g., quartz, feldspar, carbonates). Unfortunately, the CL signal originating from organic matter is not only of low intensity but also decreased following interaction with the electron beam. A variety of beam conditions were tested to optimize intensity while minimizing beam damage (Fig. 5). For a specific accelerating voltage, to a first approximation the parameters controlling CL signal loss during analysis are beam current, dwell time, and pixel size [~ current density * time, (i_d_*t)]. At a 10 kV accelerating voltage we found an i_d_*t value of ~ 3 × 10^6^ electrons/pixel to work well for this analytical setup. As an example, for a 1024 × 768-pixel image at 250 nm/pixel (250 × 200 µm field of view), ~ 2 µm beam interaction depth, 1.5 nA of beam current, and a 300 µs dwell time, (~ 282 s) provided sufficient intensity (if present) for publication quality images, while only slightly altering the CL response. Importantly, significantly shorter imaging times (20 s) were sufficient to distinguish OM types. Electron beam interaction depth was modelled using Casino v 2.51, and a composition of C_55_H_50_NO_2_S; (84%C, 6%H, 2%N, 4%O, 4%S by weight) for organic matter.

### Image analysis

Regions of interest (ROI) on individual organic matter types in CL and fluorescence PNG images were evaluated in Igor Pro 9 software, with pixel statistics exported to spreadsheet software for manipulation. Average pixel intensity in the RGB channels was summed and used to calculate percentages of red, green, and blue emission for each ROI. The total pixel intensity for RGB channels was used to normalize other maceral types to telalginite CL and fluorescence emission.

## Data Availability

The datasets generated and/or analyzed during the current study are available in the ScienceBase repository^51^ (10.5066/P9FEZVJ6) and are included in this published article.
